# Differences in Anxiety Levels of Various Murine Models in Relation to the Gut Microbiota Composition

**DOI:** 10.3390/biomedicines6040113

**Published:** 2018-12-04

**Authors:** Eunchong Huang, Shinwon Kang, Haryung Park, Soyoung Park, Yosep Ji, Wilhelm H. Holzapfel

**Affiliations:** Department of Advanced Green Energy and Environment, Handong Global University, Pohang-si, Gyeongbuk 37554, Korea; hec1324@gmail.com (E.H.); shinwonk@naver.com (S.K.); haryung@microbes.bio (H.P.); soyoung@microbes.bio (S.P.); yosep@microbes.bio (Y.J.)

**Keywords:** gut microbiota, probiotics, modulation, anxiety, host genetics

## Abstract

Psychobiotics are probiotic strains that confer mental health benefits to the host through the modulation of the gut microbial population. Mounting evidence shows that the gut microbiota play an important role in communication within the gut–brain axis. However, the relationship between the host genetics and the gut microbiota and their influence on anxiety are still not fully understood. Hence, in our research, we attempted to draw a connection between host genetics, microbiota composition, and anxiety by performing an elevated plus maze (EPM) test on four genetically different mice. Four different breeds of 5-week-old mice were used in this experiment: Balb/c, Orient C57BL/6N, Taconic C57BL/6N, and Taconic C57BL/6J. After 1 week of adaptation, their initial anxiety level was monitored using the EPM test via an EthoVision XT, a standardized software used for behavorial testing. Significant differences in the initial anxiety level and microbial composition were detected. Subsequently, the microbiota of each group was modulated by the administration of either a probiotic, fecal microbiota transplantation, or antibiotics. Changes were observed in host anxiety levels in correlation to the shift of the gut microbiota. Our results suggest that the microbiota, host genetics, and psychological symptoms are strongly related, yet the deeper mechanistic links need further exploration.

## 1. Introduction

With an estimated total population of 10^14^ microorganisms, the human gut microbiota represent a complex and vast ecosystem exceeding the total number of human cells by more than 10 times [1]. This heterogeneous microbial population of the human gastrointestinal tract (GIT) comprises a highly diverse prokaryotic microbiome, with the Bacteroidetes, Firmicutes, Actinobacteria, Proteobacteria, and Verrucomicrobia as the major bacterial phyla. Within these phyla, the Bacteroidetes and Firmicutes accommodate the highest species abundancy, constituting more than 90% of the human gut microbiota [2,3]. These two phyla significantly influence the host by their involvement in human metabolism, nutrition, and immunomodulation. The gut microbiome and the role it plays in human physiology has been (and still is) the subject of extensive research. Recent reports strongly suggest the association of an imbalance in the normal gut microbiota (dysbiosis) with conditions such as inflammatory bowel disease (IBD) [4,5], obesity [6,7], type II diabetes [8,9], and colorectal cancer [10].

It has been reported that an altered gut microbiota may contribute to the development of psychological disorders such as anxiety and depression [11]. Thus, the microbiome–gut–brain axis is an acknowledged entity that has recently been coined to portray probiotics that may have a medical advantage for patients experiencing mental illness [12]. It should be noted that several probiotic microorganisms produce and/or sense neurochemicals from the host [13]. Moreover, a large body of literature documents that modulation of the gut microbiome can alter the production of certain bacterial metabolites such as butyrate, and this metabolite appears to improve brain function by preventing neurodegeneration and promoting regeneration [14].

Recent research of the human microbiome has shown that the composition of the microbiota may depend on the host’s health, genetics, and environmental location. Increasing evidence from the literature emphasizes the importance of the gut microbiome as a modulator of the crosstalk between diet and the development of metabolic dysfunction [15]. Moreover, depending on the genetic background of the host, it may induce different changes in the gut microbiota and create diverse interactions with the microbiome. Thus far, only a few studies have performed a comparative genomic and phenotypic analysis of different mouse strains [16], showing that differences in single nucleotide polymorphisms and the composition of the microbiota can result in differences in the outcome [17].

In this article, we highlight how different breeds of mice and three different C57BL/6 substrains with differences in the gut microbiome may explain the different degrees of anxiety in different individuals. We show how treatment with a probiotic strain (*Lactobacillus rhamnosus* GG) and fecal microbiota transplantation (FMT) can impact on the host’s anxiety in correlation with the ratio of Firmicutes and Bacteroidetes. Finally, we analyzed the butyrate production by fecal samples to determine the potential role of butyrate as a key bacterial metabolite in the relationship between the gut microbiome and host anxiety.

## 2. Experimental Section

### 2.1. Bacterial Strains and Culture Conditions

The probiotic test strain, *Lactobacillus rhamnosus* GG (LGG), was grown in MRS broth (Difco Laboratories Inc., Franklin Lakes, NJ, USA). A fresh culture was prepared daily for feeding during the intervention period by growing to reach the late log phase, collecting by centrifugation at 16,000× *g* for 5 min at 4 °C, and washing twice with PBS (phosphate buffered saline). The culture was suspended in 200 µL of PBS and orally administered at a level of 2.0 × 10^9^ colony forming units (CFU)/mouse/day.

### 2.2. Animal Experiments

The animal study was approved by the Handong Global University ethical committee in Korea on 16 June 2016 (HGUIACUC20160616-002). Five-week-old C57BL/6NTac (Tac 6N), C57BL/6NCrljOri (Ori 6N), and C57BL/6JBomTac (Tac 6J) male mice and BALB/cAnNTac (Balb/c) mice were purchased from Koatec (Gyeonggi, Korea). All the mice were acclimated with an identical chow diet in a controlled environment (at 23 ± 1 °C and 55 ± 10% humidity, in a 12 h light/dark cycle) with free access to filtered water. After acclimation, their initial anxiety was measured using the elevated plus maze test (EPM).

After measuring the initial anxiety level, experimental animals were divided as shown in Supplementary Figure S1. Ori 6N PBS and Tac 6N PBS represented the control group with PBS administration, while Ori 6N LGG and Tac 6N LGG received *Lactobacillus rhamnosus* GG (LGG) and represented the probiotic group. On the other hand, Balb/c Cont and Tac 6J Cont were the control groups with distilled water administration, and Balb/c Anti and Tac 6J Anti were the groups receiving antibiotics. Groups that were treated with the antibiotics received the cocktail of 50 mg/kg body weight (BW) of vancomycin, 100 mg/kg BW of neomycin, 100 mg/kg BW of metronidazole, and 1 mg/kg BW of amphotericin-B by oral gavage, and 500 mg/500 mL of ampicillin was added to the drinking water [18]. The administration was continued for four weeks, and each week, the anxiety levels were determined and fecal samples collected. After 5 weeks of administration, the probiotic groups were sacrificed, but the antibiotic groups were given two more weeks for the fecal microbiota transplantation (FMT). 

To investigate the effect of gut modulation by FMT, we followed the protocols used by Seekatz et al. [19]. Briefly, mice received FMT via oral gavage twice a day for two weeks. Eight fecal pellets were homogenized in 1.5 mL of prereduced PBS, and a 200-μL portion of the mixture was administered to each animal by oral gavage. Fecal samples were exchanged within the groups. The Balb/c Anti group received fecal samples of the Tac Cont group and was designated Balb/c FMT. The Tac 6J Anti group received fecal samples of Balb/c Cont group and was labelled Tac 6J FMT.

### 2.3. Elevated Plus Maze Test

Anxiety of the mice was measured according to the protocols given by the Nature protocol [20]. Briefly, before the testing, the mice were exposed to the behavioral testing room for at least 30 min. After exposure to the testing environment, the mice were placed on the center of the EPM facing toward an open arm. When the mice are placed on the maze, the video-tracking system starts and a timer is set for 5 min. The video-tracking system automatically records the respective number of entries onto the open and closed arms and the time spent on each arm. After 5 min of testing, mice were removed from the plus maze and placed back on their home cage outside the testing environment. Then, the EPM was cleaned with 70% ethanol before the next test.

The maze, made from an acrylic material, comprises four arms, of which two are open (no walls) and the other two are enclosed (15 cm high). Each arm is 30 cm long and 5 cm wide. The height of the maze is elevated 40 cm off the ground. The video-tracking system and data acquisition and analysis software we used was from EthoVision (Noldus Information Technology, Utrecht, The Netherlands).

### 2.4. Fecal Microbiota Analysis

Fecal bacterial genomic DNA was extracted using a QIAamp^®^ DNA Mini Kit (QIAGEN, Valencia, CA, USA) after the mechanical disruption of microbial cell walls. In total, 500 mg of each fecal sample was suspended in 600 μL of stool lysis buffer (Buffer ASL) in a screw cap microtube (Sarstedt, Germany) containing 0.5 g of zirconium/silica beads (size 0.1 mm) (Biospec, Bartlesville, OK, USA). This was followed by bead beating in a mini-beadbeater-16 (Biospec) for 3 min. For further isolating, washing, and eluting processes, the manufacturer’s protocols were followed. Quantitative PCR (qPCR) was performed with a SYBR Premix EX Taq (Tli RNaseH Plus) kit in an Applied Biosystems StepOnePlus™ Real-Time PCR System Thermal Cycling Block (Applied Biosystems, Foster City, CA, USA). The qRT-PCR was carried out in a heated lid at 110 °C, with the sequence of 3 min at 95 °C, 8 cycles of 95 °C for 30 s, 55 °C for 30 s, 72 °C for 30 s, and 72 °C for 5 min, and finally a hold at 4 °C used for the PCR reaction.

The fecal microbiota was analyzed using the gut low-density analysis (GULDA) method as originally introduced and validated by Bergström and coworkers in 2012 [21]. The qPCR primers used for this analysis were U1 (Universal bacteria primer), forward: ACTCCTACGGGAGGCAGCAGT, reverse: GTATTACCGCGGCTGCTGGCAC; F1 (Firmicutes primer), forward: TGAAACTYAAAGGAATTGACG, reverse: ACCATGCACCACCTGTC; and B1 (Bacteroidetes primer), forward: GGARCATGTGGTTTAATTCGATGAT, reverse: AGCTGACGACAACCATGCAG [21].

### 2.5. Analysis of Short-Chain Fatty Acids (SCFA) Using Gas Chromatography

The standard curve and the peak detection retention time for the SCFAs were analyzed with the volatile fatty acid mixture (ultrapure) from Sigma (Supelco). SCFAs were extracted from the fecal samples and analyzed against the standard curve. SCFA extraction was performed according to Schwiertz et al. [22]. Briefly, 0.1 mol/L of oxalic acid and 40 mmol/L of sodium azide were mixed with a maximum of 80 mg of sample, extracted, and incubated at room temperature for an hour in a shaking incubator. The supernatant was extracted by centrifuging at 16,000× *g* at 24 °C for 5 min. SCFAs were analyzed using a Shimadzu GC2010 and a flame ionized detector (FID) instrument. A HP INNO-WAS 39 m × 32 mm was used with a splitter temperature of 260 °C, and columns were analyzed at 100 °C to 180 °C at a rate of 27.1 psi and 25 °C/m.

### 2.6. Statistical Analysis

Graphs are presented using the GraphPad Prism 7 Program (v 7.03, GraphPad Software Inc., San Diego, CA, USA). All the data are presented as the means ± SD and were analyzed with by Fisher’s Least Significant Difference (LSD) test. Significance was considered at *p* < 0.05.

## 3. Results

### 3.1. Initial Anxiety and Gut Microbiota Composition of Substrains of C57BL/6 Mice

**Figure 1:**
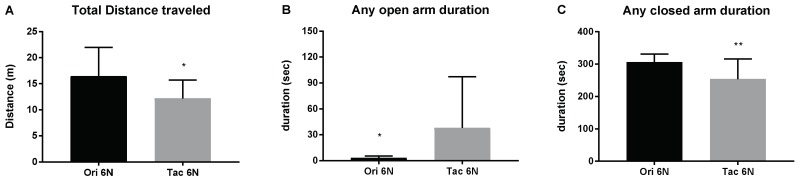
Differences in the initial anxiety of substrains of C57BL/6 mice. The initial anxiety levels of C57BL/6NCrljOri (Ori 6N) and C57BL/6NTac (Tac 6N) mice were measured through the elevated plus maze test. Each group comprised 9–14 animals. (**A**) The total distance traveled on the elevated plus maze; (**B**) Time spent on any of the open arms; (**C**) Time spent on any of the closed arms. Data are presented as the means ± SD analyzed by Fisher’s LSD test. Significance is indicated when * *p* < 0.05, ** *p* < 0.01.

Using the EPM test, the initial anxiety of substrains of C57BL/6 mice was measured. Without any treatments, the Ori 6N and Tac 6N mice exhibited different anxiety levels (Figure 1). The total distances traveled by mice in the EPM test differed among the substrains. Ori 6N mice traveled more than Tac 6N mice (Figure 1A). On the other hand, Ori 6N mice spent less time in any of the open arms and more time in any of the closed arms (Figure 1B,C). In addition, Ori 6N mice exhibited higher anxiety levels compared to Tac 6N mice.

**Figure 2:**
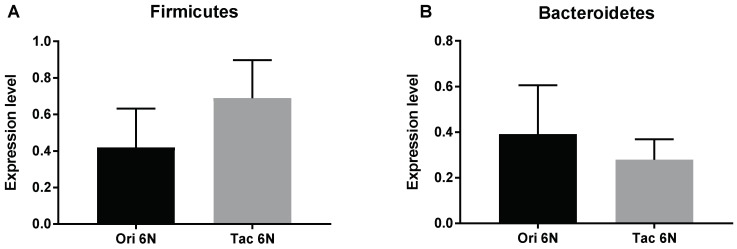
Differences in the microbiota composition of substrains of C57BL/6 mice at the onset of the experiment. The initial microbiota composition of C57BL/6NCrljOri (Ori 6N) and C57BL/6NTac (Tac 6N) mice was measured. The expression level of both graphs was compared with the Universal bacterial primer (U1). In total, 5 samples of each group were analyzed. (**A**) Expression levels of Firmicutes of the Ori 6N and Tac 6N groups; (**B**) Expression levels of Bacteroidetes of the Ori 6N and Tac 6N groups. Data are presented as the means ± SD analyzed by Fisher’s LSD test.

The two phylogenetically similar mice exhibited differences in fecal microbiota composition. Ori 6N mice contained lower levels of Firmicutes and higher levels of Bacteroidetes compared to the Tac 6N mice (Figure 2).

### 3.2. Initial Anxiety and Gut Microbiota Composition of Different Species of Mice

**Figure 3:**
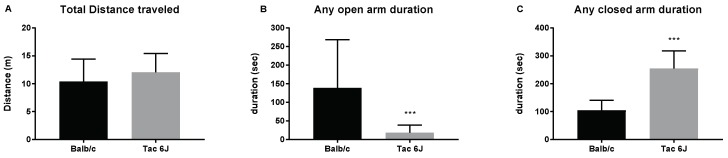
Distances traveled on the elevated plus maze and time spent in the open and closed arms. Using the elevated plus maze test, the initial anxiety of BALB/cAnNTac (Balb/c) mice and C57BL/6JBomTac (Tac 6J) male mice was measured. Each group comprised 9–14 animals. (**A**) Total distance traveled on the elevated plus maze; (**B**) Time spent on any of the open arms; (**C**) Time spent on any of the closed arms. Data are presented as the means ± SD analyzed by Fisher’s LSD test. Significance is indicated when * *p* < 0.05, ** *p* < 0.01, and *** *p* < 0.001.

Again, using the EPM, the initial anxiety levels of two different species were measured. Without any treatments, Tac 6J mice exhibited higher anxiety levels compared to Balb/c mice, whereas no significance was observed in the total distance traveled by both mice in the maze (Figure 3A). However, the duration in any of the open arms was reduced (Figure 3B), while it was higher for Tac 6J mice in any of the closed arms compared to Balb/c mice (Figure 3C).

**Figure 4:**
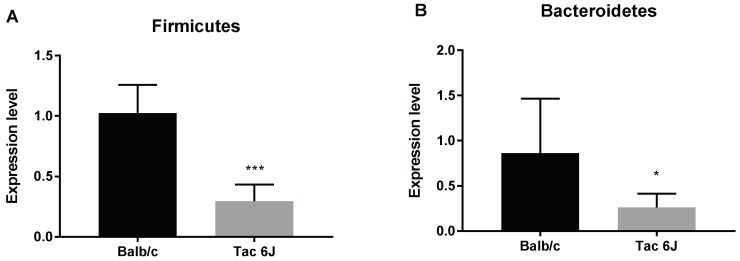
Differences in microbiota composition of different breeds of mice. Initial microbiota composition of BALB/cAnNTac (Balb/c) and C57BL/6JBomTac (Tac 6J) mice was measured through quantitative real-time PCR analysis. The expression level of both graphs was compared with the Universal bacteria primer (U1). The number of samples analyzed was 5. (**A**) The expression levels of Firmicutes of Balb/c and Tac 6J; (**B**) The expression levels of Bacteroidetes of the Balb/c and Tac 6J groups. Data are presented as the means ± SD analyzed by Fisher’s LSD test. Significance is indicated when * *p* < 0.05 and *** *p* < 0.001.

The fecal microbiota of the two different species had significantly different Firmicutes/Bacteroidetes ratios. The Tac 6J mice exhibited significantly lower numbers of Firmicutes (Figure 4A) and Bacteroidetes compared to Balb/c mice (Figure 4B).

### 3.3. Microbiota Modulation through Probiotics Can Alter the Anxiety Levels, Microbiota Composition and Production of SCFAs

**Figure 5:**
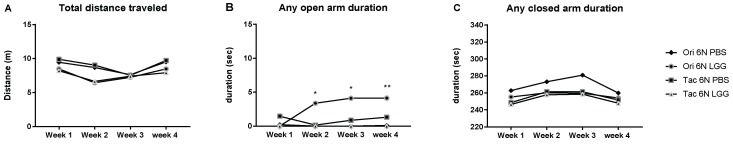
Modulation of the gut microbiota by probiotics can alter the anxiety. Probiotics reduced the anxiety levels of the experimental mice. Ori 6N PBS and Tac 6N PBS mice were both administered with 200 μL of PBS, while Ori 6N LGG and Tac 6N LGG mice both received 200 μL of *Lactobacillus rhamnosus* GG (LGG) twice a day for four weeks. Each group comprised 7 animals. (**A**) The total distance traveled on the elevated plus maze in each week; (**B**) Time spent on any of the open arms; (**C**) Time spent on any of the closed arms. The data are represented by the means and were analyzed by Fisher’s LSD test and compared to the Ori 6N LGG group. Significance is indicated when * *p* < 0.05, and ** *p* < 0.01.

After measuring the initial anxiety levels of the substrains of the C57BL/6 mice, they were divided into groups to determine possible alteration mechanisms of their initial anxiety levels. Ori 6N PBS and Tac 6N PBS mice received 200 μL of PBS, and Ori 6N LGG and Tac 6N LGG mice 200 μL of LGG twice a day for four weeks. The modulatory effect of probiotic administration on the gut microbiota and concomitant lowering influence on the anxiety of the mice was investigated by administering LGG for a period of four weeks. A significant difference in the anxiety levels of the Ori 6N LGG group could be detected even after 2 weeks, yet no effect on the Tac 6N groups could be measured (Figure 5B). The total distance traveled and duration in any of the closed arms did not differ significantly between the groups (Figure 5A,C).

**Figure 6:**
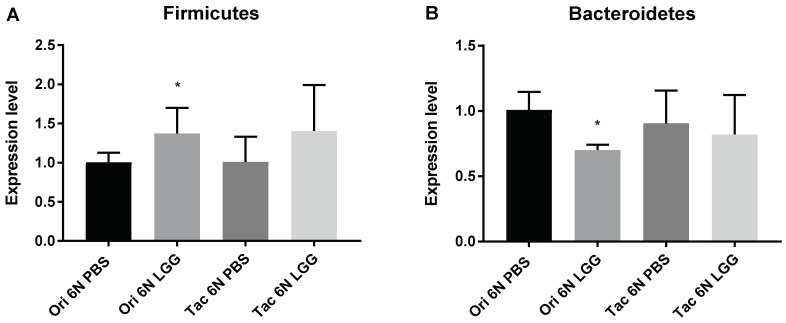
Modulation of fecal microbial composition by probiotic (LGG) administration, as compared to controls receiving PBS. Microbiota composition was measured through quantitative real-time PCR analysis. The expression level of both graphs was compared with the Universal bacterial primer (U1). The number of samples was 5–7. (**A**) Expression levels of Firmicutes in the Ori 6N and Tac 6N groups; (**B**) Expression levels of Bacteroidetes of the Ori 6N and Tac 6N groups. In the statistical analysis, Ori 6N LGG was compared with Ori 6N PBS, and Tac 6N LGG was compared with Tac 6N PBS. The data represent the means ± SD and were analyzed by Fisher’s LSD test compared to the corresponding PBS group. Significance is indicated when * *p* < 0.05.

Since LGG administration to the Ori 6N LGG and Tac 6N LGG groups resulted in significant differences in anxiety in the fourth week, the fecal microbiota composition was analyzed at that stage. The Firmicutes and Bacteroidetes composition was modulated through the administration of the probiotic LGG strain. The numbers of Firmicutes were increased in the Ori 6N LGG group, and the numbers of Bacteroidetes decreased in the Ori 6N LGG group compared to the Ori 6N PBS group (Figure 6). On the other hand, no significant changes were measured in the Tac 6N LGG group compared to the Tac 6N PBS group (Figure 6).

**Figure 7:**
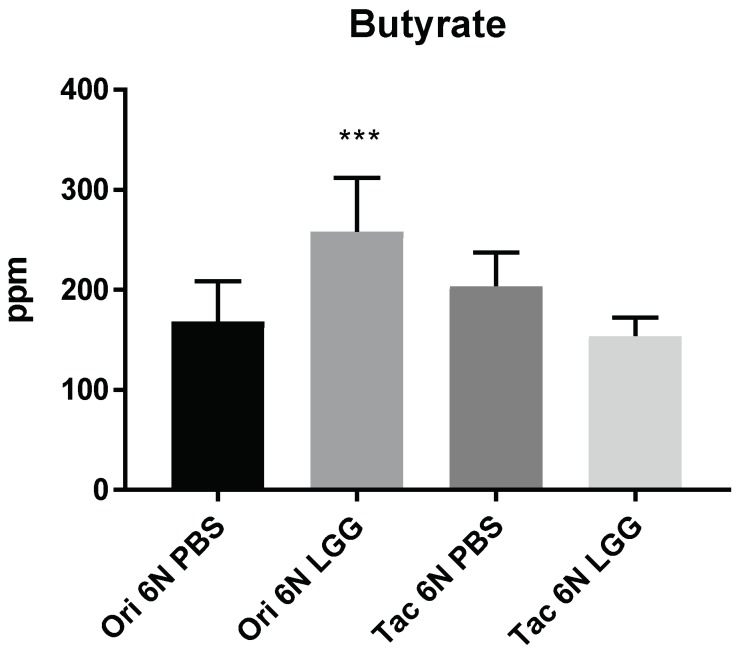
Butyrate production in the fecal samples after probiotic (LGG) treatment (compare also with Figure 5 and Figure 6). The amount of butyrate in the fecal sample was measured by gas chromatography. The detection units for the butyrate are given as parts per million (ppm). Ori 6N PBS and Tac 6N PBS groups received 200 μL of PBS, while 200 μL of LGG were administered to Ori 6N LGG and Tac 6N LGG groups twice a day for four weeks. For each group, 4–5 fecal samples were analyzed. The data are represented as the means ± SD and were analyzed by Fisher’s LSD test compared to the Ori 6N PBS group. Significance is indicated when * *p* < 0.05, ** *p* < 0.01, and *** *p* < 0.001.

The bacterial metabolites such as acetate, propionate, and butyrate from the fecal samples were measured, but no significant changes in the amount of acetate and propionate (data not shown) could be determined. However, the abundance of butyrate was differed among the groups. Only the Ori 6N LGG group showed a significant increase in the amount of butyrate (Figure 7). In summary, under the LGG probiotic feeding, phylogenetically similar mice exhibited significantly different anxiety levels, modulation of the Firmicutes/Bacteroidetes composition, and an increase in butyrate production concomitantly with reduced anxiety levels.

### 3.4. Microbiota Modulation through an Antibiotic Cocktail Can Alter the Anxiety and Microbiota Composition

**Figure 8:**
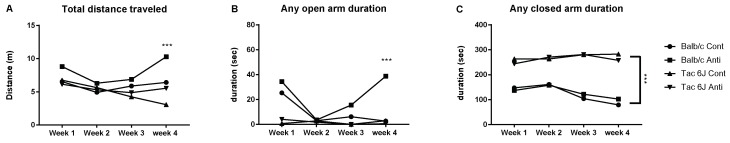
Alteration of anxiety levels by modulation of microbiota through an antibiotic cocktail. Balb/c Cont and Tac 6J Cont mice received 200 μL of distilled water, while 200 μL of an antibiotic cocktail was administered to Balb/c Anti and Tac 6J Anti mice twice a day for four weeks. Each group comprised 5–7 animals. (**A**) The total distance traveled on the elevated plus maze per week; (**B**) Time spent on any of the open arms; (**C**) Time spent on any of the closed arms. The data in Figure 8A,B are presented as the means and were analyzed by Fisher’s LSD test compared to the Balb/c Anti group. The data in Figure 8C represents the means and were analyzed by Fisher’s LSD test compared to the Balb/c and the Tac 6J group. Significance is indicated when * *p* < 0.05, ** *p* < 0.01, and *** *p* < 0.001.

The same experiment as before was conducted with different mice breeds. The animals were divided into groups to determine how their initial anxiety levels could be altered. The Balb/c Anti and Tac 6J Anti groups received 200 μL of the antibiotic cocktail and the Balb/c Cont and Tac 6J Cont mice 200 μL of distilled water twice a day for four weeks. The modulatory effect of the antibiotic cocktail on the gut microbiota and the resulting lowering of anxiety levels were investigated by feeding the antibiotic cocktail for four weeks. A significant difference was detected in the anxiety levels of the Balb/c Anti group, but no effect on the Tac 6J groups could be determined (Figure 8A–C). A significant influence was observed in the fourth week when the Balb/c Anti group traveled longer than any of the other groups on the elevated plus maze and spent most time in any of the open arms (Figure 8B). In addition, the Balb/c Anti group showed significantly less anxiety levels compared to the Tac 6J Anti groups.

### 3.5. Fecal Microbiota Transplantation Can Reverse the Anxiety and Production of SCFAs

Knowing that the modulation of the gut microbiota with antibiotics affects host anxiety, we intended to determine whether fecal microbiota transplantation can reverse host anxiety. The initial fecal samples of each group were exchanged within the groups after the administration of antibiotics. Fecal samples were orally administered for two weeks and the host anxiety level was measured with EPM.

**Figure 9:**
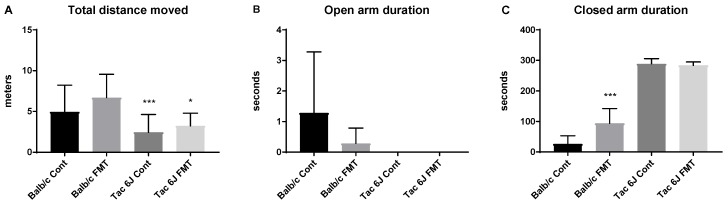
Anxiety of Balb/c and Tac 6J groups after fecal microbiota transplantation (FMT). Using the elevated plus maze test, the anxiety levels of the Balb/c and Tac 6J groups were measured. The Balb/c Anti group received fecal samples of the Tac Cont group and was labelled Balb/c FMT. The Tac 6J Anti group received fecal samples of the Balb/c Cont group and named Tac 6J FMT. The FMT samples were orally gavaged for two weeks. Each group comprised 4–7 animals. (**A**) The total distance traveled on the elevated plus maze; (**B**) Time spent on any of the open arms; (**C**) Time spent on any of the closed arms. The data are represented as the means ± SD and were analyzed by Fisher’s LSD test compared to the corresponding control group. Significance is indicated when * *p* < 0.05, ** *p* < 0.01, and *** *p* < 0.001.

The results showed that the Tac 6J Cont and Tac 6J FMT groups traveled less than the Balb/c groups, with no duration in the any of the open arms, and only remained in the closed arms. On the other hand, the Balb/c FMT group showed a reversed anxiety level after the FMT administration (Figure 9). The Balb/c FMT animals showed decreased duration spent in the open arms and a significant increase in the duration spent in the closed arms (Figure 9).

**Figure 10:**
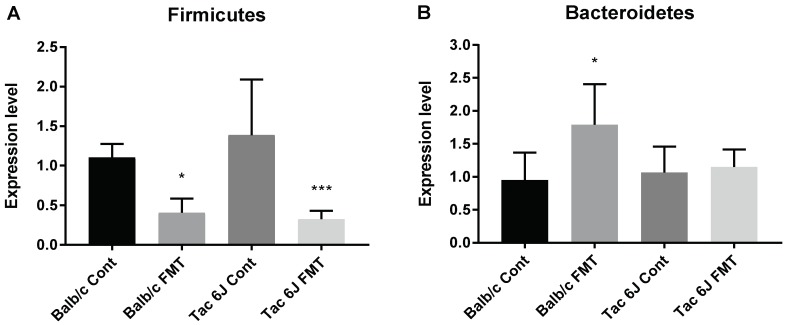
Fecal microbiota composition after FMT. Fecal microbiota composition was measured after two weeks of FMT. The Balb/c Anti group received fecal samples of Tac Cont group and was labelled Balb/c FMT. The Tac 6J Anti group received fecal samples of the Balb/c Cont group and was named Tac 6J FMT. FMT samples were orally gavaged for two weeks. Each group comprised 4–7 animals. (**A**) Expression levels of Firmicutes of the Ori 6N and Tac 6N groups; (**B**) Expression levels of Bacteroidetes of the Ori 6N and Tac 6N groups. The data represent the means ± SD and were analyzed by Fisher’s LSD test compared to the corresponding control group. Significance is indicated when * *p* < 0.05, and *** *p* < 0.001.

The fecal microbiota composition was measured and showed that FMT application resulted in the modulation of its composition. The abundance of the Firmicutes was significantly decreased in the FMT groups. While no significant changes were observed in the abundance of the Bacteroidetes of the Tac 6J FMT group, a significant increase was measured only in the Balb/c FMT group (Figure 10).

**Figure 11:**
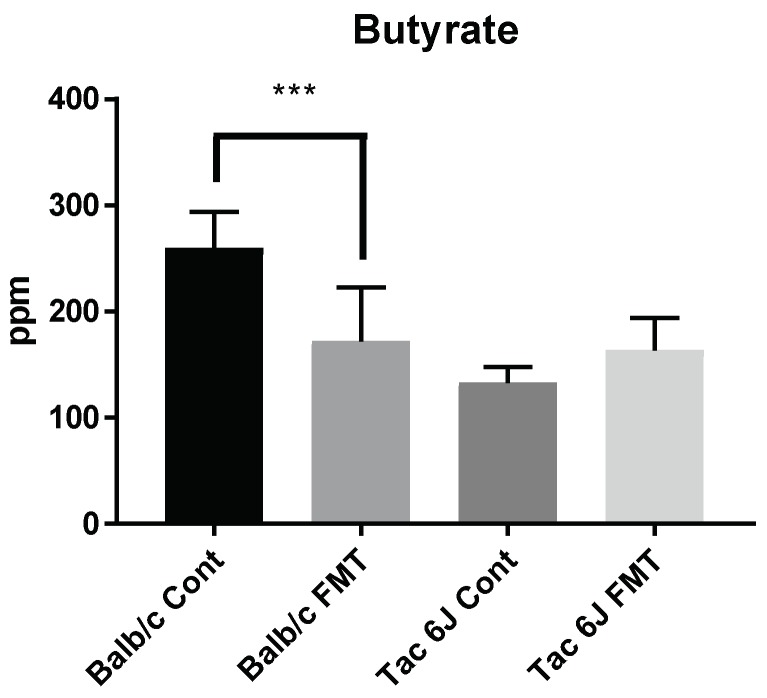
Butyrate production in fecal samples after FMT. The amount of butyrate in the fecal samples was measured by gas chromatography. The detection units for the butyrate is given as parts per million (ppm). The Balb/c Anti group received fecal samples of the Tac Cont group and was named Balb/c FMT. The Tac 6J Anti group received fecal samples of the Balb/c Cont group and was labelled Tac 6J FMT. FMT samples were orally gavaged for two weeks. Each group comprised 4-5 samples. The data represent the means ± SD and were analyzed by Fisher’s LSD test compared to the Balb/c Cont group. Significance is indicated when * *p* < 0.05, ** *p* < 0.01, and *** *p* < 0.001.

Measuring the production of SCFAs in the fecal samples showed significantly decreased amounts of butyrate in the Balb/c FMT group compared to the Balb/c Cont group. No changes were detected in the Tac 6J FMT groups (Figure 11). In summary, it appears that the increased anxiety through FMT may be correlated with a low production of butyrate in the fecal samples. Thus, as we observed different anxiety levels in different breeds of mice, the Firmicutes and Bacteroidetes composition was modulated by the antibiotic treatment, resulting in a decrease in butyrate production concomitantly with an increase in anxiety levels.

## 4. Discussion

It is expected that genome-wide and microbiome approaches for identifying regulators of anxiety-like behavior in animal models will strongly move to also include the field of psychobiotics. Revealing of and understanding potential pathways and mechanisms involved in the bidirectional communication between the gut microbiota and host behavior is making rapid progress. An imbalance in the gut microbiota, also referred to as dysbiosis, reflects the disruption of gut homeostasis and has been associated with behavioral and neurophysical deficits, including autism and depression [23,24]. Present research for the treatment of psychiatric disorders therefore deliberately involves approaches directed at the restoration of gut microbial homeostasis. However, the contribution of the host genetics in shaping the gut microbiota composition is not yet clearly understood and remains a subject of ongoing debate. Thus, by using a murine model, we intended to investigate the influence of several inbred genetic backgrounds and determine how genetic interaction and microbiota can result in different outcomes, such as measured by anxiety levels.

Differences in single nucleotide polymorphisms (SNPs) and the composition of the microbiota can result in different outcomes [17], and when comparing the 1427 SNPs, eleven different SNPs between the C57BL/6J and C57Bl/6N substrains have been reported [16]. Knowing that these differences result in different outcomes, we found that different mouse breeds and even different substrains of mice showed differences in initial anxiety and microbiota composition. Modulation through probiotics, antibiotics, and FMT can affect the host’s anxiety. Reduced anxiety was observed concomitantly with an increase in butyrate production. Substantially more research is required for understanding the intricate relationship between the quantitative and qualitative composition of the host’s microbiota, on the one hand, and anxiety levels concomitantly with increased (microbial) butyrate production. Still, these appear to be promising interventions that will probably be used in the future in conjunction with conventional pharmacological approaches. Data accumulated in recent years support the notion that anxiety levels in stressed populations may be reduced by probiotics [24,25].

The role of the microbiome in the bidirectional communication and its interaction with the host genome have been shown to be related, both in short- and long-term behavioral developments, to changes in the microbiota composition [26]. Deeper investigations into potential dysbiotic microbial patterns are still lacking, and more studies are needed on diverse genetic backgrounds and the microbiome in order to precisely identify those interactions underlying anxiety behavior. Such studies should involve multiomics approaches, by which key mechanisms underlying the regulation of the gut–brain axis may be revealed.

## Supplementary Materials

The supplementary materials are available online at http://www.mdpi.com/2227-9059/6/4/113/s1.
