# Supplementary material for: Differences in Anxiety Levels of Various Murine Models in Relation to the Gut Microbiota Composition
Source: Biomedicines. 2018 Dec 4;6(4):113. doi: 10.3390/biomedicines6040113 (PMC6315404; doi:10.3390/biomedicines6040113)
Supplement: Supplementary file 1 [file biomedicines-06-00113-s001.pdf]

Supplementary File

Differences in Anxiety Levels of Various Murine Models in Relation to the Gut Microbiota Composition

Eunchong Huang, Shinwon Kang, Haryung Park, Soyoung Park, Yosep Ji and Wilhelm H. Holzapfel*

Department of Advanced Green Energy and Environment, Handong Global University, Pohang-si, Gyeongbuk 37554, Korea; hec1324@gmail.com (E.H.); shinwonk@naver.com (S.K.); haryung@microbes.bio (H.P.); soyoung@microbes.bio (S.P); yosep@microbes.bio (Y.J.);

* Correspondence: wilhelm@woodapple.net; Tel.: +82-10-9455-1360

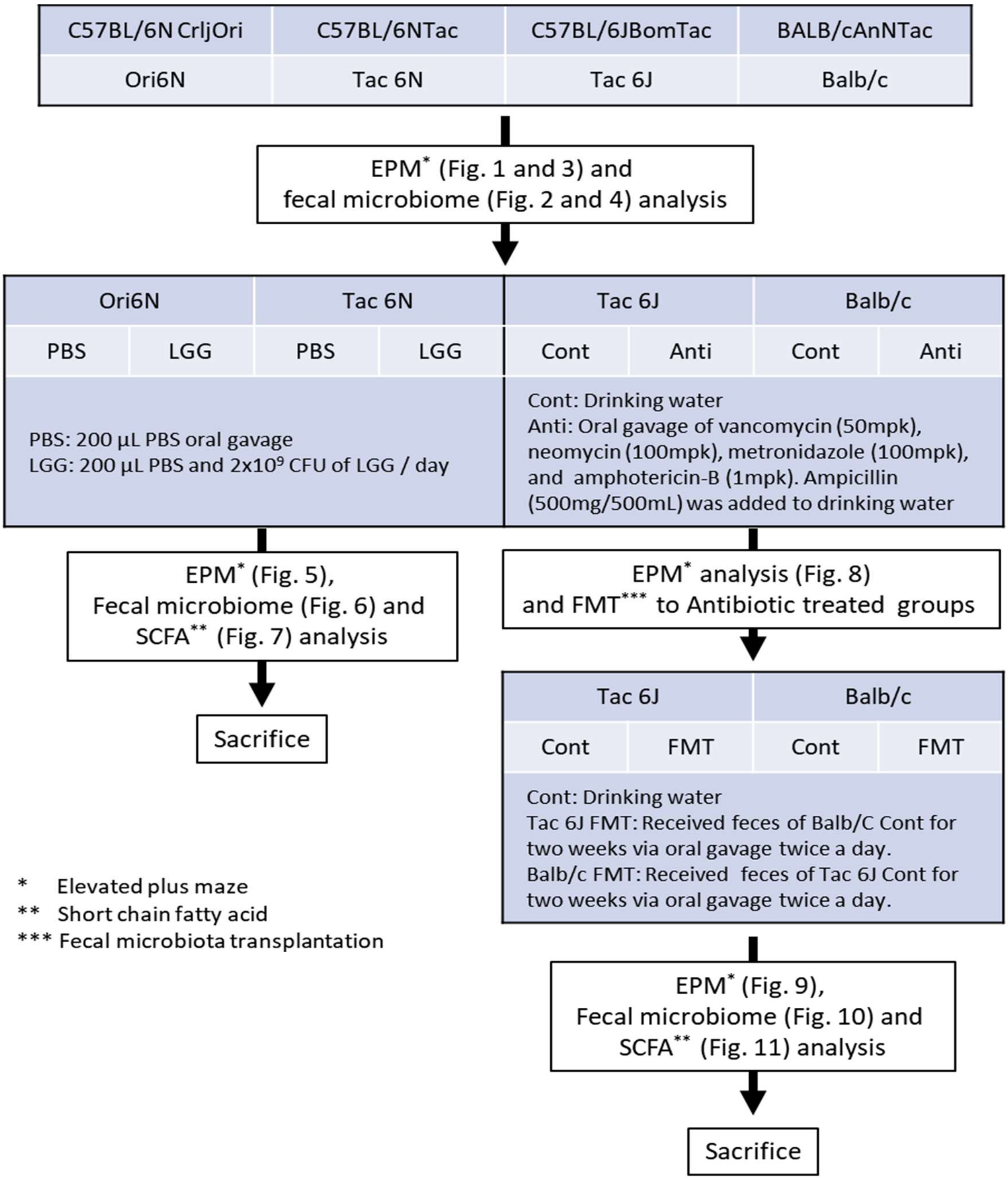

Supplementary Figure S1. Experimental scheme
